# Supplementary material for: Making a Decision between Acute Appendicitis and Acute Gastroenteritis
Source: Children (Basel). 2020 Oct 11;7(10):176. doi: 10.3390/children7100176 (PMC7650746; doi:10.3390/children7100176)
Supplement: Supplementary file 1 [file children-07-00176-s001.zip › children-943812-supplementary.docx]

**Table S1.** Laboratory data in the appendicitis group and the gastroenteritis group.

|  | **Enterocolitis group N=82** | **Appendicitis group N=32** | ***p*-value** |
| --- | --- | --- | --- |
| WBC (10^3^/uL) | 11.69 ± 4.99 | 19.07 ± 7.37 | <0.0001 |
| Hemoglobin (g/dL) | 13.18 ± 1.47 | 12.58 ± 1.4 | 0.0241 |
| Platelet count (10^3^/mm^3^) | 263.77 ± 70.69 | 324.19 ± 122.83 | 0.015 |
| Segment (%) | 75.95 ± 11.43 | 79.60 ± 8.95 | 0.14 |
| Band (%) | 0.80 ± 2.6 | 0.8 ± 1.46 | 0.0364 |
| Lymphocyte (%) | 14.99 ± 8.49 | 11.72 ± 6.56 | 0.0548 |
| ANC | 9180.59 ± 4805.87 | 15405.54 ± 6158 | <0.0001 |
| AST | 25.99 ± 7.68 | 25.16 ± 7.02 | 0.6584 |
| ALT | 19.16 ± 13.47 | 16.32 ± 7.56 | 0.4058 |
| CRP | 6.5 ± 6.43 | 15.75 ± 9.63 | <0.0001 |

WBC = white blood cell; ANC = absolute neutrophil count; AST =aspartate aminotransferase (U/L), ALT =alanine aminotransferase (U/L); CRP = C-reactive protein (10 mg/L).
